# Potential of resveratrol in the treatment of interstitial lung disease

**DOI:** 10.3389/fphar.2023.1139460

**Published:** 2023-04-06

**Authors:** Rongxiu Huo, Xinxiang Huang, Yanting Yang, Yang Yang, Jinying Lin

**Affiliations:** Department of Rheumatology and Immunology, Guangxi Academy of Medical Sciences, The People’s Hospital of Guangxi Zhuang Autonomous Region, Nanning, China

**Keywords:** interstitial lung disease, resveratrol, signal transduction pathway, immune cells, adverse reaction

## Abstract

Interstitial lung disease (ILD) is a heterogeneous group of diseases characterized by lung injury caused by lung fibroblast proliferation, interstitial inflammation, and fibrosis. Different cell signal transduction pathways are activated in response to various proinflammatory or fibrotic cytokines, such as IL-6, and these cytokines are increased in different ILDs. The overexpressed cytokines and growth factors in ILD can activate TGF-β/Smad2/3/4, NF-κB, and JAK/STAT signal transduction pathways, promote the activation of immune cells, increase the release of pro-inflammatory and pro-fibrotic factors, differentiate fibroblasts into myofibroblasts, and promote the occurrence and development of ILD. This finding suggests the importance of signal transduction pathways in patients with ILD. Recent evidence suggests that resveratrol (RSV) attenuates excessive inflammation and pulmonary fibrosis by inhibiting the TGF-β/Smad2/3/4, NF-κB, and JAK/STAT signal transduction pathways and overactivation of immune cells. In this review, advances in lung protection and the underlying mechanisms of RSV are summarized, and the potential efficacy of RSV as a promising treatment option for ILD is highlighted.

## Introduction

**FIGURE 1:**
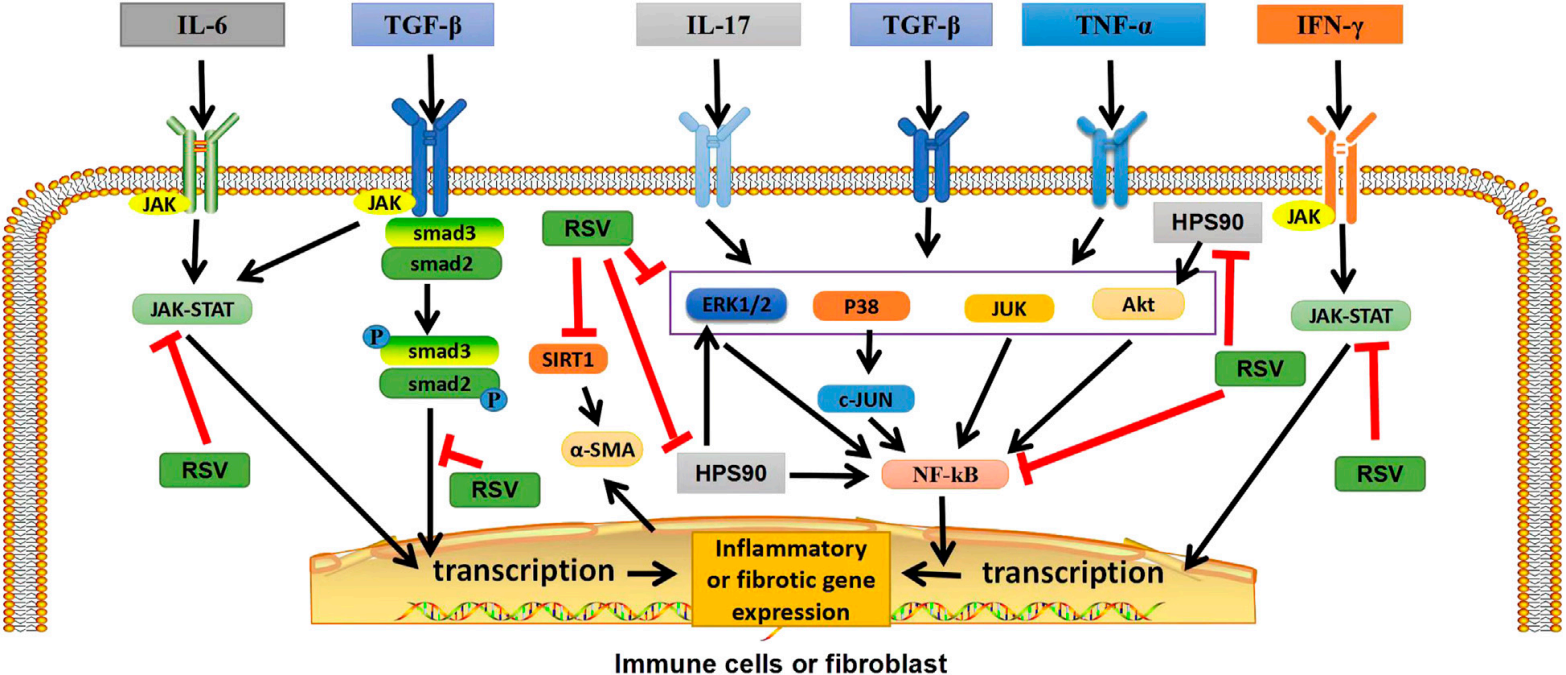
Graphical abstract: Potential targets of RSV in ILD. In immune cells or fibroblasts, RSV exerts anti-inflammatory and antifibrotic effects by inhibiting TGF-β/Smad2/3/4, NF-κB, and JAK/STAT pathways, as well as by regulating heat shock protein 90, inhibiting Akt, ERK1/2 and NF-κB pathways. In addition, RSV can also inhibit SIRT1, reduce *α*-ASM activation, and play an antifibrotic role. TGF-β, transforming growth factor β; IFN-γ, interferon-γ; TNF-α, tumor necrosis factor; P, phosphate; JAK, janus tyrosine kinase; STAT, signal transducers and activators of transcription; ERK1/2,extracellular signal-regulated protein kinases; AKT, serine-threonine kinase; NF-κB, nuclear factor-kappa B; SIRT1, sirtuin1; *α*-SMA, *α*-smooth muscle actin. “ê” indicates activation, and “—׀” indicates inhibition.

Interstitial lung disease (ILD) includes a group of heterogeneous diseases, which can be derived from a variety of different etiology, such as infection, drug, radiation-induced lung diseases and autoimmune diseases such as rheumatoid arthritis (RA), etc., leading to damage of alveolar epithelium and lung parenchyma, eventually leading to lung inflammation and fibrosis (Shao et al., 2021). ILD was characterized by inflammation or fibrosis of different degrees in the lung parenchyma. For example, in inflammatory diseases, the histological form is characterized by institutional pneumonia or non-specific interstitial pneumonia, while diseases dominated by fibrosis are characterized by common interstitial pneumonia (Mikolasch et al., 2017) (Figure1).

ILD is one of the most common and serious lung diseases. It was found that the total incidence of ILD was 19.4/100,000, and the most common diagnosis was sarcoidosis (42.6%) (Duchemann et al., 2017). Overactivation of immune cells in ILD patients resulted in the release of pro-inflammatory/pro-fibrotic cytokines and oxidative damage, and abnormal myofibroblast activation was observed with enhanced epithelial-interstitial transformation (EMT) and the secretion of large amounts of collagen for post-injury repair (You et al., 2015; Gouda and Bhandary, 2018). This leads to excessive deposition of extracellular matrix (ECM) and the development of interstitial fibrosis (Pardo and Selman, 2002). Eventually, symptoms such as a dry cough, shortness of breath and fatigue. The management and treatment of ILD remains challenging for clinicians. Currently, common therapeutic strategies include glucocorticoids and immunosuppressants (such as cyclophosphamide) (Shao et al., 2021), but sometimes they are resistant to the above-mentioned drugs and have serious adverse reactions (Huo et al., 2022). Therefore, it is urgent to find a drug with significant efficacy and few side effects.

Resveratrol (RSV) is a natural plant antitoxin, which is abundant in a variety of plants such as grapes and peanuts as well as a variety of commercial products, such as grape juice and red wine (Truong et al., 2018). Resveratrol exists in two isomeric forms, cis-trans and trans-trans, but trans is the main form, which has the most effective therapeutic benefits due to its low steric hindrance of the side chain (Cardile et al., 2007; Weiskirchen and Weiskirchen, 2016; Raj et al., 2021). Trans can be reconstituted from yeast extracts and become more biologically active forms with higher stability (Chen et al., 2007; Camont et al., 2009). It has been reported that RSV can slow down the progression of autoimmune diseases, such as RA, systemic lupus erythematosus (SLE), psoriasis (PsO) and inflammatory bowel disease (IBD), etc. (Oliveira et al., 2017). In addition, studies have shown that RSV has inhibitory effects on inflammation and anti-fibrosis, and thus has a therapeutic prospect for pulmonary diseases and fibrosis diseases (Vargas et al., 2016). At present, more and more studies have reported that RSV can play an anti-fibrosis role by inhibiting the molecules and cells in some signal transduction pathways. In animal model studies, RSV has been shown to improve ILD in mice by inhibiting the expression of Smad and Smad7, inhibiting the over-activation of immune cells, inhibiting inflammation and the proliferation and differentiation of lung fibroblasts, and reducing collagen deposition (Chávez et al., 2008; Liu et al., 2010). Recent studies have found that RSV can also play a beneficial role in the later stage of pulmonary fibrosis by regulating the metabolism of collagen in lung tissue and reducing its deposition in lung interstitium (Wang et al., 2021). In addition, by inhibiting the activation of NF-κB in macrophages and lymphocytes, the expression levels of nitric oxide and tumor necrosis factor-α (TNF-α), interleukin (IL-1β), IL-6, transforming growth factor-β (TGF-β) and TNF in pulmonary fibrosis are reduced to prevent oxidative damage (Yahfoufi et al., 2018; Fu et al., 2020). RSV may inhibit pro-inflammatory factors and down-regulate oxidative stress levels by inhibiting epithel-mesenchymal transformation and down-regulating NF-κB and TGF-β1/smad3 signaling pathways (Wang et al., 2022). At the same time, the autophagy process and the nucleotide-binding domain and leucine-rich repeat protein 3 inflammasome activities are inhibited, and the production of pro-inflammatory factors is reduced, thus improving lung inflammation and fibrosis (Ding et al., 2019). In addition, in the fusion process of autophagosome and lysosome, RSV can relieve their inhibitory state and reverse the destruction of autophagosome—lysosome fusion, thus improving ILD (Bao et al., 2022). These findings suggest that RSV can be used as a potential treatment for ILD (Figure 1).

In this review, we focus on the protective effects and potential mechanisms of RSV against ILD. Meanwhile, this review summarizes the progress of RSV in the treatment of ILD and its related adverse reactions, providing new insights for the treatment of ILD.

## Mechanism of ILD formation

### Immune cells and ILD

In ILD patients, especially those with autoimmune ILD, immune disorders can activate macrophages, T cells, B cells, and NK cells to damage the alveolar epithelium, and then repeated repair leads to the gradual destruction of functional lung parenchyma, which is replaced by increased deposition of non-functional connective tissue (Chambers and Mercer, 2015), eventually leading to ILD. The mechanism of different types of immune cells in ILD is shown in Table 1.

**TABLE 1:** Mechanism of different immune cells in ILD.

Immune cells	Effects in ILD	Mechanisms of different immune cells in ILD
Macrophages	Differentiated into “pro-inflammatory” classic M1 macrophages or “pro-fibrotic” M2a macrophages in ILD Kishore and Petrek (2021). Secreting cytokines and chemokines Kishore and Petrek (2021)	Activating PI3K/AKT, TGF-β/Smad, p38 MAPK, ERK1/2, and JAK/STAT pathways Kishore and Petrek (2021). Produces pro-inflammatory and pro-fibrotic cytokines Wills-Karp and Finkelman (2008); Van Dyken and Locksley (2013). Cytokines:TNF-α, IL-1, IL-4, IL-6, IL-10, IL-12, IL-23, IL-33, IFN-γ, TGF-β, et al. ↑ Kishore and Petrek (2021). Chemokines:CCL2, CCL18, CCL24 MCP-1, MIP-1α, et al. ↑ Kishore and Petrek (2021). Cytokines and chemokines are involved in lung inflammation and pulmonary fibrosis Kishore and Petrek (2021). In addition, inducing the differentiation of fibroblasts into myofibroblasts Wang et al. (2020). Induced epithelia to proliferate Wang et al. (2020)
T cells	Differentiated into Th1, Th2, Th9, and Th22 cells in ILD Deng et al. (2023). Secreting cytokines Deng et al. (2023)	Cytokines: IL-4, IL-5, IL-9, IL-13, IL-17A, IL-22, TGF-β, TNF-α, et al. ↑ Wynn (2008); Desai et al. (2018). Activating PI3K/AKT, TGF-β/Smad, ERK1/2 and JAK/STAT pathways. Activating fibroblasts and myofibroblasts Deng et al. (2023). Extensively promotes ECM production and collagen deposition Deng et al. (2023). Extracellular matrix proteins, types I and III collagen and fibronectin increased in ILD Roberts et al., 2003
B cells	Increased B cell activation and an increased number of memory B cells and plasmablasts in ILD and secreted antibodies and cytokines Heukels et al. (2019); Roman and Chiba (2021)	Cytokines: IL-6, IL-8, BAFF, et al. ↑ Neys et al. (2021), Roman and Chiba (2021). Activating JUK, p38 MAPK, and mTOR pathways Roman and Chiba 2021. Autoantibodies produced are closely associated with ILD Lee et al. (2013), including anti-citcitline protein antibody (ACPA), rheumatoid factor (RF), Sjogren’s syndrome-associated antigen A antibody, anti-synthetase antibody or anti-melanoma differentiation-associated protein 5 antibody, anti-topoisomerase I antibody, Anti-U11/U12 ribonucleoprotein antibodies and anti-eukaryotic initiation factor 2B antibodies and so on
NK cells	The number of NK cells and their activity in ILD decreased, and CD56 and CD16 were expressed simultaneously Aquino-Galvez et al. (2009); Cruz et al. (2021). Secreting cytokines. Cruz et al. (2021)	Cytokines: IFN-γ↑, TNF-α↑, TGF-β↓ Cruz et al. (2021). MTOR signaling, JNK, IL-8, and IL-23 signaling were upregulated in ILD Huang et al. (2021). Cytokine imbalance promoted the development of ILD. Expressed higher levels of granzyme B, the most potent NK-cell cytotoxic enzyme Cruz et al. (2021)

Symbols: ↑, up-regulation; ↓, down-regulation.

### Macrophages and ILD

Macrophages are immune cells that differentiate from monocytes and play a role in the balance between innate and adaptive immunity. In the process of fibrosis caused by immune imbalance, monocytes in the blood are recruited into the lungs and activated into macrophages under the action of chemokines, and cytokines are secreted to differentiate fibroblasts into myofibroblasts. In addition, macrophages can be polarised into “pro-inflammatory” classical M1 macrophages that secrete pro-inflammatory and/or pro-fibrotic cytokines, such as interleukin (IL) -1β, or “pro-fibrotic” M2a macrophages that secrete pro-fibrotic cytokines (Kolahian et al., 2016; Bellamri et al., 2019), thus promoting the progression of pulmonary fibrosis. Especially in SSc-ILD. In inflammatory environment, promoting nuclear factor (NF) -κB and Janus kinase (JAK)/signal transductor and transcription activator (STAT) pathways in macrophages to induce the production of pro-inflammatory factors, such as tumor necrosis factor (TNF) -α, IL-1, IL-6, etc. (Balachandran and Adams, 2013), can promote the occurrence of ILD.

### T cells and ILD

At present, preclinical studies have determined that some T cell subtypes are associated with fibrosis, such as Th2 and Th17, which promote fibrosis. Up-regulation of Pd-1 on CD4^+^ T cells increases type 1 collagen synthesis and promotes pulmonary fibrosis through STAT 3-mediated IL-17A and TGF-β production (Celada et al., 2018). At present, the most studied ILD are IPF and SSc-ILD. The two diseases share similar common features, such as T cell profiles (Th2, Th17, increased ratio of CD4 to CD8 T cells), T cell cytokine profiles (IL-4, IL-5, IL-10, and IL-17 of IPF, and IL-4, IL-5, IL-6, IL-10, IL-13, and IL-22 in SSc-ILD (Bagnato and Harari, 2015). In RA-ILD, like IPF and SSc-ILD, the expression of IL-17 receptor was up-regulated, indicating that Th17 cell-mediated immunity was involved in the pathogenesis of ILD (Wang et al., 2019; Zhang et al., 2019). Furthermore, the number of CD4^+^ T cells in lung tissues of RA-ILD patients was significantly higher than that of idiopathic UIP patients, suggesting that immune dysregulation may be more prevalent in RA-ILD patients than in idiopathic UIP patients (Turesson et al., 2005). These results indicated that the imbalance of T cell subsets might be involved in ILD formation.

### B cells and ILD

As a result of immune disorders in patients with autoimmune diseases, autoantigens are presented to CD4^+^ helper T cells by antigen-presenting cells and then further presented to B cells, so that B cells differentiate into plasma cells and produce and secrete autoantibodies. Recently, several studies have pointed to a possible role of B cells in IPF, RA, or ILD associated with connective tissue disease. Some studies have found that, compared with healthy people, the phenotype distribution of B cells in the peripheral blood of IPF patients is abnormal, and the percentage of plasmabytes in IPF patients is negatively correlated with forced vital capacity (Xue et al., 2013). The authors detected focal aggregates of CD20-positive B cells in all IPF lung tissues using immunohistochemistry (Xue et al., 2013). Atkins et al. (2006) compared the lung biopsy specimens of patients with RA-ILD and control subjects and observed obvious follicular B cell hyperplasia in patients with RA-ILD. Recent studies have also found that the total peripheral blood B cell count is higher in patients with RA-ILD, but the frequency of memory B cells is lower. The mechanism may be that some of them selectively migrate to the lung tissue (Shimizu et al., 2021). In some ILDs, B cell production of antibodies may play a key role. For example, there is an association between antibodies, including anti-citrullinated protein antibodies (ACPA) and rheumatoid factor (RF), and the risk of RA-ILD (Katsumata et al., 2019). The presence of Sjogren’s syndrome-related antigen A antibody is a predisposing factor for ILD in patients with Sjogren’s syndrome (Flament et al., 2016). Anti-synthetase antibody or anti-melanoma differentiation-associated protein 5 (MDA5) antibodies are associated with myositis-related ILD, while anti-topoisomerase I (anti-scl70), anti-U11/U12, and anti-eukaryotic initiation factor 2B antibodies are associated with SSc-ILD (Kuwana et al., 2021). These results suggest that B cell-formed autoantibodies may be involved in the formation of ILD.

### Natural killer (NK) cells and ILD

Previous studies have shown that NK cells play a key role in the pathogenesis of acute lung injury (Liu et al., 2016). There are two different NK environments in human pulmonary fibrosis: one in the lung and the other in the peripheral blood. In pulmonary fibrosis, NK cells are thought to counteract the fibrotic activity of TGF-β by producing the anti-fibrotic mediator interferon-γ (IFN-γ), thereby inducing anti-fibrotic signals in the lung (Culley, 2009). As for NK cells in the peripheral blood, studies have found that the percentage and absolute number of NK cells in the peripheral blood of 11 IPF patients are both high (Esposito et al., 2005). In addition, a retrospective study found that the absolute and relative counts of CD3^−^CD56^+^ NK cells in the peripheral blood of patients with RA-ILD were higher than those of patients with RA, whereas the percentages of T cells and CD4^+^ T cells were lower, suggesting that the occurrence of RA-ILD may be unbalanced with the lymphocyte subsets of patients. In particular, CD3^−^CD56^+^ NK cells are associated with T cell imbalances (Lai et al., 2019). The increase in the percentage and absolute number of CD3^−^CD56^+^ NK cells clearly suggests a possible involvement in the pathogenesis of human ILD and suggests a novel approach for studying ILD. These results suggest that NK cells may be involved in the formation of profibrotic, antifibrotic, and ILD conditions; however, further studies are needed.

### Cytokine and growth factor-related signal transduction pathways and ILD

**FIGURE 2:**
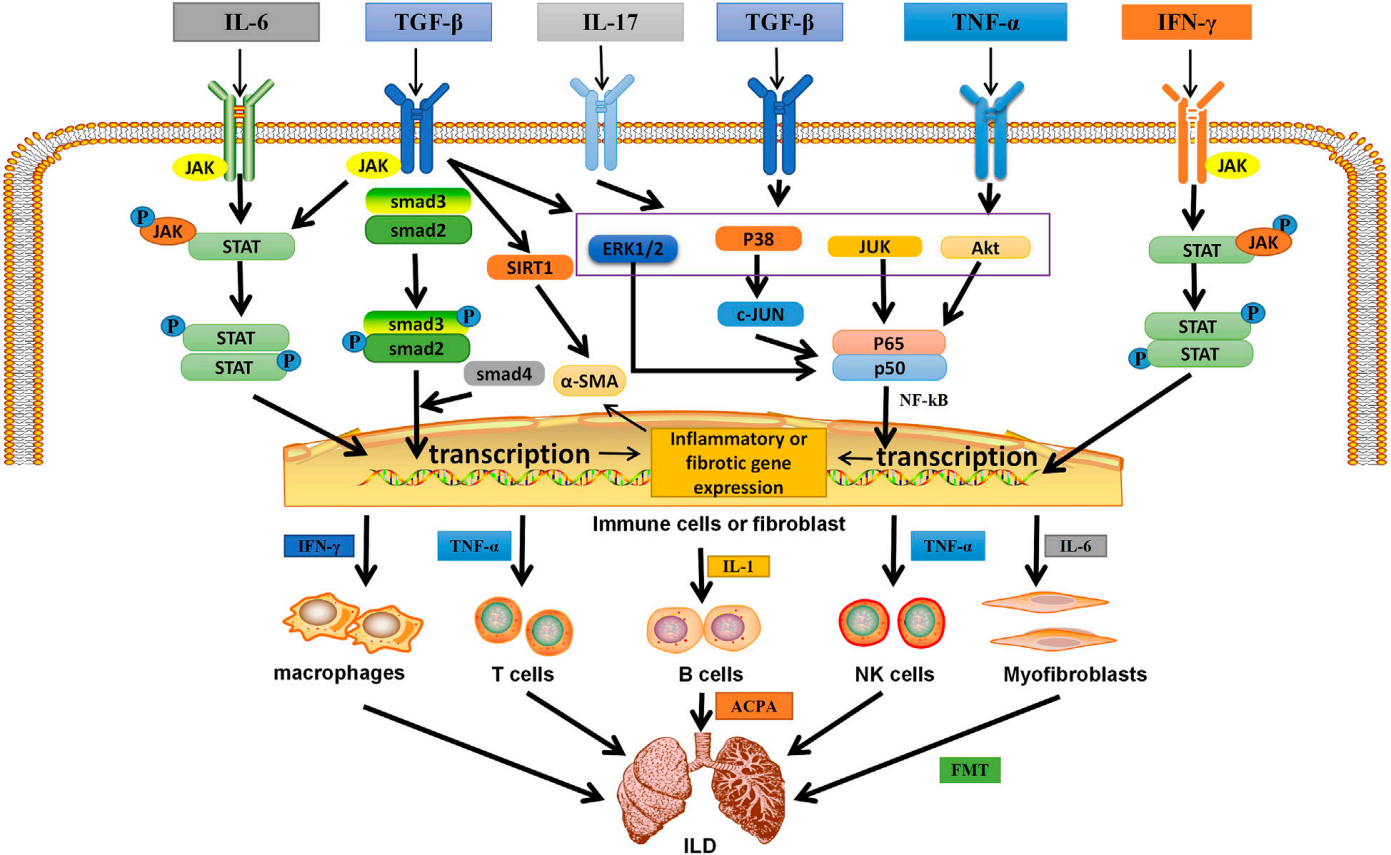
The related mechanism of ILD occurrence. Cytokines and growth factors, after binding to their corresponding receptors in immune cells or myofibroblasts, can be activated through different signal transduction pathways. Activated immune cells can secrete a variety of cytokines. They further activate macrophages; T, B, and NK cells; and myofibroblasts. They are over-activated and can secrete pro-inflammatory and pro-fibrotic cytokines, which is the environment for the formation of inflammation and fibrosis in the lung. Meanwhile, in different autoimmune diseases, B cells secrete corresponding antibodies, which are related to pulmonary fibrosis. In addition, IL-6 can activate myofibroblasts, causing them to undergo EMT and form ECM, which is deposited in the lung. Eventually ILD is formed. IL, interleukin; TGF-β, transforming growth factor β; IFN-γ, interferon-γ; TNF-α, tumor necrosis factor; P, phosphate; JAK, janus tyrosine kinase; STAT, signal transducers and activators of transcription; ERK1/2,extracellular signal-regulated protein kinases; AKT, serine-threonine kinase; NF-κB, nuclear factor-kappa B; SIRT1, sirtuin1; *α*-SMA, *α*-smooth muscle actin; ACPA, anticitrullinated peptide antibodie; ECM,extracellular matrix; EMT, epithelial-mesenchymal transition; ILD,Interstitial lung disease.

Multiple molecular pathways are activated by pro-inflammatory/pro-fibrotic cytokines (e.g., IL-6, IL-17, and TNF-α) and growth factors (e.g., TGF-β), which are increased in different ILD. The above overexpressed cytokines and growth factors in ILD were activated in the corresponding intracellular signal transduction pathways, such as the Smad-dependent and Smad-independent signaling pathways, NF-κB and JAK/STAT signaling pathways, which were closely related to the occurrence and development of ILD (Figure 2 **)**.

① Activation of a Smad-dependent signaling pathway: after TGF-β induction, the downstream transduction molecule Smad2 binds to Smad3, which is subsequently phosphorylated and activated to form trimers with Smad4 and transmit signals from the cell membrane to the nucleus (Feng and Derynck, 2005).

② Activation of the Smad independent signaling pathway and the NF-κB pathway: TGF-β receptor complex or TNF-α interacts with tumor necrosis factor receptor-associated protein 6 (TRAF6) to promote its own ubiquitination (Tzavlaki and Moustakas, 2020). TRAF6 activates TGF-β-activated kinase 1 (TAK1) through Lys63-linked polyubiquitination, which in turn is activated by phosphorylating MAP kinase kinases (MKK) MKK4, MKK3, and MKK6. MKKS further activate their downstream kinases JNK and p38, which can then phosphorylate their target transcription factors (TF) and regulate transcription (Tzavlaki and Moustakas, 2020). In addition, TGF-β-induced ShcA tyrosine phosphorylation promotes Ras protein activation. This leads to the sequential activation of Raf, mitogen activated protein kinase, and extracellular signal-regulated protein kinase (ERK1/2). Activated ERK1/2 phosphorylates TF. TGF-β also promotes the activation of rapamycin C2 through phosphatidylinositol 3-kinase (PI3K), which further recruits and phosphorylates serine/threonine kinase (AKT) (Tzavlaki and Moustakas, 2020), thereby activating the NF-kB pathway. Studies have shown that TGF-β can also induce Sirtuin 1 activation and activate *α*-smooth muscle actin (α-SMA) (Ma and Li, 2020). In addition, IL-17 forms heterodimer complexes with its receptors IL-17RA and IL-17RC to recruit NF-κB activator 1(Act1) through SEFIR domain interactions. Subsequently, Act1 binds to TRAF6 *via* its TRAF6 binding site, recruiting TRAF6 into the IL-17R complex) (Zhu and Qian, 2012). Act1 acts as E3 ubiquitin ligase to polymerize TRAF6. TRAF6 also acts as an E3 ubiquitin ligase, which may ubiquitinate TAK1 complex and activate PI3K/AKT leading to NF-κB activation (Zhu and Qian, 2012). In addition, heat shock protein (HSP) is also involved in ILD formation. HSP90 has been shown to directly regulate ERK through dissociation of the ERk-HSP90-CDC37 complex (Erazo et al., 2013), thereby activating the ERK pathway. HSP90 also regulates AKT, and activation of AKT pathway has been reported in different cells with different pathological functions for pulmonary fibrosis (Colunga Biancatelli et al., 2020).

③JAK/STAT signaling pathway: Activation of the JAK/STAT pathway begins with the binding of ligands (usually cytokines, growth factors, such as IL-6, TGF-β, and IFN-γ) to their receptors, inducing JAK dimerization. The receptor-associated JAK is then activated and phosphorylates the tyrosine residue in the tail of its receptor cell to form p-JAK. These phosphorylations act as docking sites for STAT and bind to them *via* its SH2 domain, which phosphorylates STAT and is activated by tyrosine phosphorylation to form p-STAT, which subsequently becomes a dimer and translocations from the cytoplasm to the nucleus, where they act as transcription factors (Montero et al., 2021). Thus, STATs are activated by JAKs and transferred from cytoplasm to nucleus, and then bind to specific DNA sequences to direct gene transcription, resulting in increased expression of pro-inflammatory cytokines TNF-α and IL-1β and pro-fibrotic cytokine IL-6 in the lung (Yang et al., 2019).

In the lung, the above cytokines and growth factors are activated in immune cells through the above signal transduction pathways, resulting in the expression of pro-inflammatory, pro-fibrosis genes and *α*-SMA (Ma and Li, 2020), thus activating more immune cells (including macrophages, T cells, B cells, and NK cells) and lung fibroblasts (Figure 2). The above pathway can up-regulate the expression of macrophage related polarization markers (such as CD86 and CD206), and cause macrophages to differentiate into “pro-inflammatory” M1 macrophages and release inflammatory factors (such as IFN-γ), and “pro-fibrotic” M2a macrophages and release pro-fibrotic cytokines (IL-6) (Lescoat et al., 2020). T cells and NK cells release pro-inflammatory cytokines (such as IFN-γ, TNF-α, IL-1, and IL-17) and pro-fibrotic cytokines (IL-6) (Figure 2) (Alesci et al., 2022). In addition to releasing cytokines, B cells can also differentiate into plasma cells and secrete ACPA, anti-SCL70 antibodies (Bellamri et al., 2019). Moreover, positive feedback amplifies the above signaling pathways and promotes the occurrence of pulmonary inflammation and fibrosis (Lescoat et al., 2020). Lung fibroblasts can induce self-proliferation or mesenchymal transformation through the pathway, resulting in abnormal expression of *α*-SMA and EMC components, thus causing fibroblasts to lose differentiation and obtain mesenchymal phenotype of myofibroblasts, ultimately leading to ILD formation (Duchemann et al., 2017) (Figure 2).

### Effects of RSV on immune cells

RSV can enter cells by a variety of means (e.g. passive diffusion, endocytosis, or *via* transporters) and bind to specific receptors, such as the integrin receptor alphavβ3 (Delmas and Lin, 2011; Ho et al., 2020). It helps regulate innate and adaptive immunity, such as macrophages, B cells, T cells, and NK cells, thereby inhibiting overactivation of cells, reducing the production of pro-inflammatory or pro-fibrotic factors, and controlling the progression of ILD. Effects of RSV on immune cells (Table 2).

**TABLE 2:** Effects of RSV on immune cells.

Immune cells	Effects of RSV on immune cells
Macrophages	Decrease COX-2 expression; Modulate NF-κB activation; Eexert antioxidant properties; Inhibit cytokine secretion, such as TNF-α and IL-6, etc. Yang et al. (2017); Chen and Musa (2021)
T cells	Inhibit T cell activation; Reduce Th17 cells; Reduce IL-17,IFN-γ and TGF-β,etc; Modulate T cell regulation Poles et al. (2021)
B cells	Inhibit TGF-β expression; Induced SIRT1; Inactivates STAT3 Lai et al. (2019)
NK cells	Effect on NK cells killing activity; Decrease cytokine expression, such as IFN-γ,TNF-α and IL-13, etc. Krzewski and Strominger (2008); Lai et al. (2019)

### RSV and autoimmune diseases

The development of autoimmune diseases may result in damage to one or more body tissues or organs. Autoimmune diseases disorders of immune cells in the body, which cause immune cells to overactivate and produce a large number of inflammatory factors, such as TNF-α, IFN-γ, and IL-1β. Overstrong immune response can simultaneously attack different organs or tissues, leading to local or systemic immune response, such as RA, amyotrophic lateral sclerosis (ALS), PsO, SLE, IBD, etc. (Oliveira et al., 2017). RSV is a well-studied substance known for its effects on a large number of chronic diseases and its numerous therapeutic benefits, including anti-regulatory immunity, anti-inflammatory, etc. In autoimmune diseases, RSV can inhibit synovial macrophages, reduce angiogenesis, leukocyte and lymphocyte recruitment, fibroblast proliferation and protease secretion, especially in RA, and thus inhibit cartilage and bone destruction at pus formation sites (Oliveira et al., 2017). It can also inhibit the differentiation of T lymphocytes into Th2 and Th17, and reduce the activation of B cells and the production of autoantibodies (Oliveira et al., 2017). Cytokines are also closely related to autoimmune diseases. For example, macrophages increase the recruitment of neutrophils at the site of inflammation through the activation of NF-κB, and produce related cytokines (IFN-γ, TNF-α, IL-17, IL-21, and IL-23, etc.). In experimental animals, When IL-6, TNF-α and other disorders occur, they are sufficient to cause destructive arthritis (Oliveira et al., 2017). RSV not only inhibited TNF-α and IL-1β-induced NF-κB activation, but also activated SIRT1 and inhibited RelA acetylation in mice with arthritis. In addition, the expression of NF-κB-induced inflammatory factors such as TNF-α, IL-1β, IL-6, metalloproteinase (MMP)-1 and MMP3 is decreased (Yamamoto and Gaynor, 2001). It can also reduce the mRNA expression levels of IL17 and IL19, reduce the thickness of animal skin and improve the damage caused by psoriasis (Malaguarnera, 2019). In addition, RSV reduced mRNA expression levels of IL-6, TNF-α, IFN-γ, JAK, and STAT when treating inflammatory mice (Yang et al., 2019). It is suggested that RSV can inhibit the anti-inflammatory effect of JAK/STAT signaling pathway. Therefore, RSV may have some efficacy in the treatment of autoimmune diseases by regulating the immune system and interfering with multiple cellular and molecular processes (see Table 3). Epithelial interstitial transformation (EMT) is an important factor in the development of pulmonary fibrosis (Tian et al., 2017). Some studies have found that the activation of NF-κB is an effective inducer of EMT activation, suggesting a close relationship between pulmonary fibrosis, inflammation and EMT (Chua et al., 2007; Chen et al., 2018a). In autoimmune diseases, some cytokines, such as IFN-γ, TNF-α, IL-17, and IL-6, are associated with ILD (Montero et al., 2021). In addition, it has been found that JAK-STAT pathway plays an important role in early alveolitis and the development of ILD (Banerjee et al., 2017). In RA-ILD mouse models, intraperitoneal injection of a JAK inhibitor (tofacitinib) improved symptoms and inhibited the progression of ILD (Sendo et al., 2019). RSV can not only reduce the levels of NF-κB and JAK-STAT, but also reduce the levels of IFN-γ, TNF-α, IL-17, and other cytokines, so RSV may have the potential to treat ILD, especially auto-immune-related ILD.

**TABLE 3:** Study on RSV in autoimmune diseases.

Diseases	The role of RSV in autoimmune diseases	Study on the correlation of resveratrol in autoimmune diseases
RA	RSV is able to act by reducing the production of autoantibodies, Th17 population, oxidative stress and NF-κB activation. Resveratrol also reduces COX2 and PGE2 expression and activates SIRT1 Yamamoto and Gaynor (2001); Tsai et al. (2017); Malaguarnera (2019)	Chen et al. (2014) reported that oral administration of resveratrol (10 or 50 mg/kg body weight) for 2 weeks reversed arthritis dysfunction in adjuvant arthritis rats. Riveiro-Naveira et al. (2016) used an acute model of antigen-induced arthritis in rats treated with resveratrol (12.5 mg/kg) daily for 2 months by oral gavage. They observed significantly reduced knee swelling, which suggests that oral administration of resveratrol can reduce severity in this model
SLE	RSV acts as an SIRT1 activator, inhibiting proliferation of B and T cells and antibody production Cardile et al. (2007)	Wang et al. (2014) used alupus BALB/c mouse model, in which the mice received an 0.5 mL injection of pristane and were treated with RSV (50 mg/kg/day and 75 mg/kg/day) over 7 months and the serum levels of autoantibodies and kidney damage were assessed. They found that resveratrol was able to attenuate proteinuria, decrease IgM and IgG kidney deposition, and reduce kidney histological lesions
PsO	Inhibiting the production of IL-17 (produced by Th17), IFN (produced by Th1) and directly inhibiting the proliferation of keratinocytesl Lynde et al. (2014)	Kjær et al. (2015) used a mouse model of imiquimod-induced psoriasis, a study demonstrated that RSV could ameliorate the damage caused by psoriasis, reducing the thickness of the animals' skins
ALS	RSV acts by activating SIRT1 and regulates its substrate expression, increases the SOD1 useful life, reduces ROS, and acts in mitochondrial biogenesis as an antioxidant and antiapoptoticl Yamamoto and Gaynor (2001)	ALS mouse model was intraperitoneally injected with RSV at the dose of 25 mg/kg body weight/day. RSV can significantly attenuate the motor neuron loss and reduce the muscle atrophy and dysfunction in the ALS micel Malaguarnera (2019)
IBD	RSV is capable of acting on the inhibition of inflammatory cells (Th1 and Th17 profiles) inflammatory cytokines (TNF-α,IL-1β) and neutralizing ROSl Cardile et al. (2007)	Martín et al. (2004) used RSV (5–10 mg/kg) gavage treatment, performed at 48, 24, and 1 h prior to the induction of colitis, resulted in improved acute experimental colitis, demonstrating a chemopreventive role of RSV in animal models 24 h later. Likewise, clinical signs of the disease-diarrhea, weight loss, and bleeding-were attenuated in the animals that received this dietl Sánchez-Fidalgo et al. (2010)

### RSV and ILD

ILD is associated with decreased lung function, and complications can lead to rapid deterioration of the clinical course of ILD patients. In the past few years, two anti-fibrosis drugs pirfenidone and Nidanib have been approved for the treatment of ILD patients, but there are associated adverse reactions, including gastrointestinal adverse reactions, fatigue, weight loss, etc. (Morrow et al., 2022). More and more studies confirm that RSV is an ideal treatment for ILD. Fagone et al. (2011) studied the effect of RSV on TGF-β-induced myoblast activation of human lung fibroblasts. They found that RSV inhibited collagen production, lung fibroblast proliferation, and *α*-SMA protein levels by inhibiting the phosphorylation of ERK1/2 and Akt, and down-regulated TGF-β1 levels by inhibiting TGF-β1/Smad2/3/4 signaling pathways, improving pulmonary fibrosis in mouse models (Fagone et al., 2011). In addition, RSV also down-regulates TGF-β-induced Smad2/3 phosphorylation by reducing the phosphorylation levels of c-Jun, *α*-SMA, p-Smad2, Smad7, and p38, significantly reducing TGF-β-induced collagen deposition and alleviating symptoms in rats (Wang et al., 2018). Recent results showed that RSV (40 mg/kg) could not only inhibit the expression of TGF-β1 in IPF rats, but also inhibit the phosphorylation of Smad2/3 and ERK1/2, significantly reduce the elevated levels of TNF-α, IL-6, and IL-13 excitability in ILD rats, and inhibit the damage of alveolar wall. Inflammatory cell infiltration, congestion and edema are reduced (Liu et al., 2020). These results indicate that RSV can inhibit TGF-β/Smad2/3/4 and NF-κB signaling pathways to play anti-inflammatory and antifibrotic roles.

In addition, Li et al. found that RSV targeted TAK1, significantly inhibited TAK1 activation, inhibited alveolar macrophages in alveoli, and reduced lung inflammation and pulmonary fibrosis in mice (Li et al., 2017). In bleomycin-induced EMT-related pulmonary fibrosis, SIRT1 expression is decreased, and the expression of type I collagen and *α*-SMA is increased (Rong et al., 2016). After RSV treatment, SIRT1 expression is increased, which reduces alveolar epithelial cell damage, fibroblast proliferation, collagen deposition and pulmonary fibrosis, and is related to lung protection (Ma and Li, 2020).

A recent study evaluated the effect of RSV on RA-ILD. We treated RA-ILD rats with RSV (10 mg/kg/day) orally for 4 weeks, and found that RSV inhibited the JAK/STAT signaling pathway (i.e. the expression of JAK and STAT were significantly reduced in lung tissue) and decreased the levels of pro-inflammatory cytokines TNF-α, IL-6, and IL-1β. Meanwhile, it can promote the production of anti-inflammatory factor IL-10 and reduce lung inflammation (Colunga Biancatelli et al., 2020). In addition, the authors found that RSV improved pulmonary pathology (reduced inflammatory cell infiltration, reduced collagen deposition, significantly thinner alveolar wall thickness) and reduced fibrosis degree (Colunga Biancatelli et al., 2020)^.^ Therefore, RSV plays an anti-inflammatory and antifibrotic role by regulating the JAK/STAT signaling pathway, thereby improving RA-ILD.

In ILD, HSP90 expression was increased, and HSP90 ATPase activity was increased in fibroblasts isolated from fibrotic lung injury (Sontake et al., 2017). HSP90 may also contribute to the development of pulmonary fibrosis through IL-6, as HSP90 mediates activation of the nuclear factor kappa light chain enhancer in the B cell (NF-κB) dependent inflammatory pathway, promoting IL-6 production (Bohonowych et al., 2014; Bellaye et al., 2018). In IPF, alveolar epithelial type II cells promote pulmonary fibrosis through HSP90-AKT signaling (Chen et al., 2021). In SSc-ILD patients, HSP90 was overexpressed, and HSP90 could promote the persistence of myoblasts in pulmonary fibrosis by enhancing TGF-β signal transduction pathway (Štorkánová et al., 2021). In RA-ILD, IFN-γ produced by T cells stimulated by anti-citrullinated HSP90 indicated a TH1 immune response, and thus participated in the development of ILD (Chen et al., 2018b). In addition, inhibition of HSP90 has an effective antifibrotic effect *in vitro* and in mouse pulmonary fibrosis models (Koh et al., 2015). The use of HSP90 inhibitors showed that inhibition of HSP90 can down-regulate the expression of AKT, ERK, and NF-κB, regulate the stability of TGF-β receptor, and interfere with the Smad and non-Smad (P-ERK) TGF-β signaling cascade. Thus, EMT is reduced and cell proliferation, formation of fibrotic mediators and ECM are reduced (Schumacher et al., 2007). Studies have shown that RSV can inhibit HSP90 (Liu et al., 2014). RSV inhibition of HSP90 can not only down-regulate MEK1/2 and ERK phosphorylation and NF-κB expression, but also reduce IL-2, IFN-γ, and TNF-α levels (Huang et al., 2020). Therefore, RSV may have similar effects as an inhibitor of HSP90. HSP90 may be the target of RSV, which can inhibit signal transduction pathways, reduce cytokine levels and play the role of anti-fibrosis, so as to achieve the purpose of treating ILD. However, there is still a lack of relevant research, and more studies are expected to further confirm. In conclusion, RSV inhibits different signaling pathways and immune cells, and therefore has therapeutic potential for treating ILD.

### Potential adverse reactions to RSV

In general, RSV ingestion is generally well tolerated. In animal models, RSV has been shown to be non-irritating and genotoxic to the skin and eyes. After 90 days of subchronic toxicity test, RSV was found to have no adverse effects on the body and no reproductive toxicity at the maximum dose of 700 mg/(kgd) (Williams et al., 2009). This preliminarily proves that RSV is non-toxic and safe. However, some studies have found that RSV has certain side effects. When rats were treated with 1.0 and 3.0 g/(kgd) RSV, different degrees of dehydration, dyspnea, nephrotoxicity and elevated serum liver enzymes were observed in female and male rats (Shaito et al., 2020). This indicates that high dose RSV has certain toxicity (Hebbar et al., 2005). When administered continuously for 4 weeks at doses of 0, 300, 1,000, and 3,000 mg/kg/day, no adverse reactions occurred at doses up to 300 mg/kg/day, whereas 1,000 and 3,000 mg/kg/day caused renal toxicity (Crowell et al., 2004). When RSV was administered simultaneously to rats at (0.3, 1.0, or 3.0 g/kg/day), abnormal expression of liver genes was noted, possibly indicating liver injury (Hebbar et al., 2005). A significant increase in bilirubin levels was observed at 1,000 (mg/kg)/day RSV in rats, but no adverse effects were observed at 200 mg/(kgd) in rats and 600 mg/(kgd) in dogs (Johnson et al., 2011). The toxicity of RSV to target organs remains to be further studied.

In human subjects, a daily RSV dose of 450 mg has been reported to be safe for a 60 kg person (Moon et al., 2004). However, some adverse effects have been reported, and high dose RSV intake appears to have negative effects on metabolic status, endothelial health, inflammation, and cardiovascular markers in human patients (Ramírez-Garza et al., 2018). For example, higher doses of RSV (1,000 mg/day) have recently been shown to increase biomarkers of cardiovascular risk (e.g., oxidized LDL, soluble intercellular adhesion molecule-1, etc.), while lower doses have no effect on the same biomarkers (Mankowski et al., 2020). In a recent meta-analysis of 18 studies included, adverse reactions occurred in two studies (Zhang et al., 2021), one of which initially administered 500 mg, qd, followed by an increase of 500 mg daily every 3 days, with a maximum dose of 3,000 mg daily (1,000 mg, tid). Adverse events occurred in three of the five patients. In the RSV group, one patient with a history of fatty liver disease developed asymptomatic and mild elevated alanine aminotransferase, another patient developed diarrhea and mild hypoglycemia, and one patient developed mild cellulitis at the biopsy site (Goh et al., 2014). In another study, which administered RSV 500 mg once daily plus losartan 12.5 mg, only two patients (one RSV and one placebo) complained of side effects of gastritis, such as mild dyspepsia, throughout the study of 30 patients in the RSV and placebo groups (Sattarinezhad et al., 2019). Therefore, more *in vivo* studies involving animal models are necessary, and more clinical trials of RSV in humans are needed to verify its efficacy and safety, especially before it can be considered for therapeutic or prophylactic use in humans.

## Conclusion

ILD is one of the most important factors that directly affect the quality of life of patients. Long-term use of immunosuppressant and antifibrotic drugs can lead to many inevitable side effects. In addition, current drug treatments are still insufficient to reduce ILD progression and mortality worldwide. Bioactive natural ingredients derived from natural herbs may provide additional benefits in the prevention and treatment of ILD and represent an important source of new drug screening and development.

RSV inhibits TGF-β/Smad, NF-κB, and JAK/STAT pathways and immune cells, reduces the levels of pro-inflammatory and pro-fibrotic cytokines, and has powerful anti-inflammatory and anti-fibrotic effects. Therefore, TGF-β/Smad, NF-κB, and JAK/STAT pathways are potential targets of RSV in the treatment of ILD. It is also a potential and beneficial candidate for combination with other clinical antifibrosis agents. In addition, Piceatannol (PIC), a derivative of RSV, promotes autophagy by inhibiting the TGF-β1-Smad3/ERK/P38 signaling pathway, resulting in a decrease in the number of activated myoblasts, significantly reducing BLM-induced collagen deposition and myoblast accumulation (Sheng et al., 2022; Tieyuan et al., 2022). It also indicated that the related derivatives of RSV and the extracted compounds may also be able to treat ILD. However, the oral bioavailability of RSV is very low, with a maximum oral bioavailability of only 20%, although the total absorption rate in the gut is as high as 70% (Walle et al., 2004). Oral and intravenous radioisotope labeling of 14C RSVS suggests that the stage of biotransformation is a rate-limiting factor in RSV bioavailability. Therefore, targeted delivery of RSV to desired tissues or increased stability of RSV *in vivo* through the development of slow release systems are critical for improving bioavailability (Santos et al., 2019; Thipe et al., 2019). In addition, RSVS have been reported to have synergistic therapeutic effects when combined with other bioactive ingredients and micronutrients, giving RSVS a more stable chemical structure, higher solubility, and easier absorption in the small intestine (Williamson and Manach, 2005)^.^

The drug dosage of RSV in the treatment of ILD is still unclear, as the dosage used in different studies varies. If clinical studies of RSV dose gradient Settings exist, deeper mechanisms of therapeutic effect may be clearly understood. Future studies should further explore the effects of different drug doses of RSV on treatment and the deeper mechanism, and open a new window for the treatment of ILD. Although the results of most studies on polyphenols have proved promising, further research on animals and humans is warranted. By extensively evaluating the biological activity, efficacy, safety and appropriate dose of RSV, and determining specific molecular targets and structure-activity relationships, it provides a new way for clinicians to treat ILD.
